# Cardiac Sympathetic Nerve Sprouting and Susceptibility to Ventricular Arrhythmias after Myocardial Infarction

**DOI:** 10.1155/2015/698368

**Published:** 2015-12-17

**Authors:** Chang-Yi Li, Yi-Gang Li

**Affiliations:** Department of Cardiology, Xinhua Hospital, Shanghai Jiaotong University School of Medicine, 1665 Kongjiang Road, Shanghai 200092, China

## Abstract

Ventricular arrhythmogenesis is thought to be a common cause of sudden cardiac death following myocardial infarction (MI). Nerve remodeling as a result of MI is known to be an important genesis of life-threatening arrhythmias. It is hypothesized that neural modulation might serve as a therapeutic option of malignant arrhythmias. In fact, left stellectomy or *β*-blocker therapy is shown to be effective in the prevention of ventricular tachyarrhythmias (VT), ventricular fibrillation (VF), and sudden cardiac death (SCD) after MI both in patients and in animal models. Results from decades of research already evidenced a positive relationship between abnormal nerve density and ventricular arrhythmias after MI. In this review, we summarized the molecular mechanisms involved in cardiac sympathetic rejuvenation and mechanisms related to sympathetic hyperinnervation and arrhythmogenesis after MI and analyzed the potential therapeutic implications of nerve sprouting modification for ventricular arrhythmias and SCD control.

## 1. Introduction

The majority of sudden cardiac deaths (SCD) are caused by ventricular tachyarrhythmias (VT) secondary to acute myocardial infarction (MI) [1], which is a major cause of morbidity and mortality in patients with MI. Accumulating evidence from basic and clinical studies has shown a close association between VT, SCD, and sympathetic activity in animals and patients with MI [2]. Following an ischemic insult, sympathetic axon fibers become dysfunctional and infracted, which is followed by neural remodeling characterized by nerve sprouting and heterogeneous patterns of sympathetic innervation [3–5]. Abnormal sympathetic innervation leads to accentuated dispersion of repolarization and increased automaticity and triggers activity, which is underlying the susceptibility to, and initiation of, malignant arrhythmias. Because of this importance, the molecular mechanisms responsible for nerve regeneration after MI, as well as exact mechanisms by which sympathetic hyperinnervation may trigger lethal arrhythmias, have received a great deal of attention from investigators. In this paper, we review current knowledge on regulatory mechanisms implicated in neural sprouting and cardiac arrhythmias following MI, which may provide new valuable therapeutic options to decrease the incidence of VT, VF, and SCD after MI.

## 2. Normal Autonomic Nervous System of the Heart

**Figure 1:**
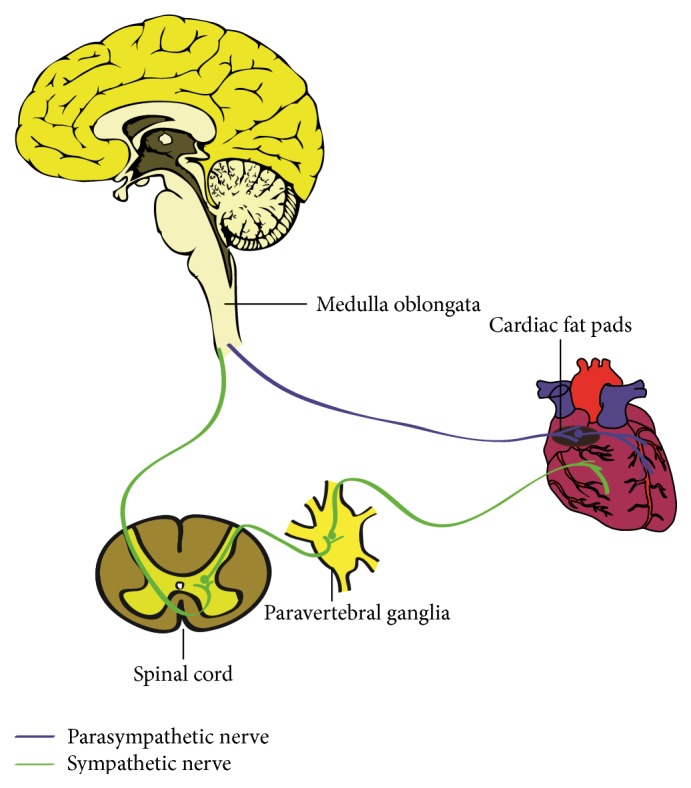
Anatomy and distribution of autonomic innervation of the heart. The cardiac parasympathetic nerves come from parasympathetic neurons located in the cardiac fat pads whose preganglionic fibers are carried within the vagus nerve. The cardiac sympathetic nerves come from the paravertebral ganglia and project from the base of the heart into the myocardium.

The heart is extensively innervated by the autonomic nervous system which is mainly composed of sympathetic and parasympathetic components (Figure 1). Sympathetic nerves come from sympathetic neurons in the superior cervical ganglia, stellate (cervicothoracic) ganglia, and thoracic ganglia which communicate with corresponding cervical or thoracic spinal cord [6]. For these postganglionic cells, axons form the superior, middle, and inferior sympathetic input and then project from the base of the heart into the myocardium along the epicardial vascular structures of the heart [7], whereas parasympathetic innervation to the heart originates predominantly in the parasympathetic neurons in cardiac ganglia whose preganglionic fibers are carried within the vagus nerve. Once entering the pericardial sac, sympathetic and parasympathetic nerve fibers together with cardiac ganglia form an exquisitely complex network to control of cardiac performance. Throughout the heart, there are multiple ganglionated plexi functions as integration centers, concentrated within fat pads and scattered over the atria and ventricles [8]. It is widely recognized that parasympathetic innervation is sparse in the left ventricle (LV) compared with sympathetic nerves [8]. A recent report, however, suggested that parasympathetic fibers innervate both atria and ventricles [9]. The density of parasympathetic and sympathetic innervation in the special conduction system such as the sinoatrial node and atrioventricular node is considerably higher than working myocardium [10, 11]. In addition to regional difference, autonomic innervation also shows some functional asymmetry. For instance, stimulation of right sympathetic nerves versus left usually results in an increased heart rate versus a prominent increase of blood pressure, respectively, although this functional asymmetry is not absolute. Thus, there might be a theory that ganglionated plexi may serve as a primary site to manage extensive signal inputs ensuing providing output to a specific cardiac structure and presenting exquisitely complex and varying functional significance [12–14].

## 3. Sympathetic Rejuvenation after MI

It has been well known that MI could result in degeneration and death of sympathetic fibers within the scar [15]. In addition, regions of sympathetic denervation also occur in the viable myocardium beyond the infarcted area [16–19]. After injury, in contrast to central neurons, peripheral neurons typically regenerate back to their targets [20, 21]. Regeneration of sympathetic nerves in the heart after MI has been well characterized in both animals and humans [3, 5, 19, 22–30]. By using growth-associated protein 43 (GAP43), a protein associated with axonal growth cone, to quantify the density of nerve fibers, Zhou and coworkers demonstrated that after MI, nerve sprouting is slow but accelerates to reach an apparent rate within 1 week and then progressively declined by 2 month [26, 27]. Compatible with this finding, ^123^I-meta-iodobenzylguanidine (MIBG) studies presented evidence that sympathetic reinnervation exists in the infracted hearts of patients [3]. Cao et al. performed immunocytochemical staining for S100 protein, neurofilament protein, and tyrosine hydroxylase on explanted hearts to study the distribution and the density of sympathetic nerves. They reported that the density of nerve fibers was significantly higher in the periphery of necrotic tissues of failed hearts [24]. There are inconsistent reports about sympathetic regeneration within cardiac scar. Li et al. found that cardiac infarct was not reinnervated after cardiac ischemia-reperfusion [19], whereas, following chronic cardiac ischemia, there is robust sympathetic regeneration within the infarct [5, 29, 30]. A recent study showed that chondroitin sulfate proteoglycans (CSPGs) presented in the infarct inhibit sympathetic axon outgrowth by acting through Protein Tyrosine Phosphatase Sigma (PTPRS) [31]. Additionally, in the heart postinfarct, sympathetic hyperinnervation is also likely to coexist with denervation [19, 32], a critical contributor to the onset of serious ventricular arrhythmias [33, 34]. Indeed, future studies are warranted to address the important and complex issues area, timing and patterns of sympathetic denervation and reinnervation, and their molecular mechanisms in the infracted heart. In conclusion, the post-MI heterogeneous of sympathetic transmission accounting for a nonuniform electrophysiologic response may create a high-yield substrate for ventricular arrhythmias [1].

### 3.1. Neurotrophic Factors Are Critical for Regeneration

In fact, the sympathetic efferent regeneration effort is triggered by neurotrophic factors, particularly nerve growth factor (NGF). NGF is a potent neural chemoattractant that exerts critical functions for the survival and differentiation of sympathetic neurons and promotes peripheral nervous axon outgrowth [35, 36]. Following MI, NGF levels significantly increased [26, 30, 37–39] which is likely a major contributor to the causes of sympathetic hyperinnervation [40, 41]. In contrast, infarct-stimulated nerve sprouting was blocked by NGF antibodies in vitro cocultures [30, 31]. Of interest, Zhou et al. observed an upregulation of cardiac NGF with a greater magnitude at the infarcted site than the noninfarcted LV free wall, which was not consistent with patterns of neurilemma proliferation [26]. Transcardiac (difference between coronary sinus and aorta) NGF concentration increased immediately after MI; however, the NGF levels significantly increased in the left stellate ganglion (LSG) from 3 days after MI, without a concomitant increase in mRNA. The authors speculated that NGF is likely transported retrogradely to LSG [42], which then triggers nerve sprouting in noninfarcted LV sites. Indeed, NGF infusion to the LSG is associated with augmentation of MI-induced sympathetic nerve sprouting [40, 43]. In addition to release from damaged cells within the heart, increased NGF content may be partly due to synthesis from cells such as cardiomyocytes [44], Schwann cells [45], and inflammatory cell [29, 30, 46]. Using transgenic and knockout mouse models, Ieda et al. have shown that ET-1 could facilitate cardiomyocytes' production of NGF during MI [44]. Considering that ET-1 is strongly induced during the MI, the ET-1/NGF pathway may contribute to nerves regeneration following MI [44]. From a mechanistic perspective, NGF might function through the p75 neurotrophin receptor and TrkA receptor in sympathetic neurons to stimulate axon outgrowth [47–50]. Signal transducer and activator of transcription 3 (STAT3) is necessary for NGF-induced sympathetic regeneration in the heart after MI [51]. These would suggest that NGF seems to have a greater impact on sympathetic regeneration in the heart after injury.

### 3.2. Sema3A Is a Axonal Chemorepellent

Although axon pruning of heart during development is critical for establishment of appropriate neural circuitry which is regulated by a number of factors such as ephrins and semaphorins [52–54], the link between sympathetic hyperinnervation and axon pruning after MI requires further studies to elucidate. Sema3A is a class 3 secreted semaphorin and identified as a potent neural chemorepellent able to cause the retraction and collapse of the neuronal growth cone in the vertebrate [55]. A bulk of evidence documented that sema3A could modulate development of major structure of central nervous system such as the brain and spinal cord and determine peripheral neural patterning and projections, and participate in axon regeneration and neural repair [56, 57]. Ieda and coworkers have shown that sema3A is strongly expressed in the heart during embryogenesis, gradually decreased after birth, which plays a negatively regulatory role in determining the density and patterning of cardiac sympathetic innervations [52]. Importantly, we found cardiac nerve injury caused by MI could not significantly trigger the reexpression of sema3A, but overexpression of sema3A in MI border zone could reduce sympathetic hyperreinnervation accompanied by reduced inducibility of ventricular arrhythmias [58]. Wen et al. demonstrated that sema3A significantly shortens monophasic action potential duration (APD) and effective refractory period at infarct border zones after MI compared with control group [59]. They speculated this alleviation of electrical remodeling after MI may be attributable to suppression of sympathetic nerve sprouting by sema3A. These results indicate that sema3A may play a role in sympathetic pruning in the peri-infarct ventricle.

Overall, as MI results in marked upregulation of neural chemoattractants expression without corresponding elevated chemorepellents expression, it might be that this unbalance in the infracted heart leads to the excessive regeneration behavior of sympathetic nerve axons, thereby contributing to the enhanced risk of ventricular arrhythmias and SCD after MI.

## 4. Neural and Electrical Remodeling after MI

MI could cause important changes in cellular electrical activity (electrical remodeling) particular of border-zone cells functionally based on ion-channel abnormalities due primarily to ionic loss, membrane breakdown, and intracellular acidosis [60]. For example, a variety of K^+^ currents such as *I*_to_, *I*_K1_, *I*_Ks_ are downregulated in border-zone cells [61–63]. Generally, sympathetic stimulation leads to shortening of APD and reducing dispersion of refractoriness [64]. In the continued presence of chromanol 293B, a specific *I*_Ks_ blocker, sympathetic stimulation produces an abbreviation of the epicardial and endocardial cells APD but not that of the M cells, resulting in an accentuated dispersion of repolarization and widening of the T wave [65]. In patients afflicted with LQT1, whose *I*_Ks_ is also abnormal, *β*-adrenergic stimulation with epinephrine could result in torsade de pointes (TdP) [66]. Thus marked abnormalities of the electrophysiologic properties of myocardium after infarct most likely distort the patterns of functional myocardial innervations, therefore enhancing the susceptibility to arrhythmias. On the other hand, sympathetic hyperinnervation at scar border zones leads to increased peak Ca^2+^ current and increased repolarization dispersion [67]. In a postinfarct model, nerves sprouting was associated with dispersion of repolarization, along with changes of outward and inward rectifier K^+^ currents [68]. Ajijola et al. demonstrated that animals with anteroapical infarcts showed altered epicardial propagation during sympathetic stimulation [69]. These data suggest heightened sympathetic tone likely make a previously dormant channel conductive (or vice versa), consequently altering the electrophysiological properties of the innervated tissues. The coupling between augmented sympathetic remodeling and electrical remodeling provides a plausible explanation for a higher risk of life-threatening arrhythmias after MI.

## 5. Sympathetic Nerve Sprouting and Ventricular Arrhythmogenesis after MI

**Figure 2:**
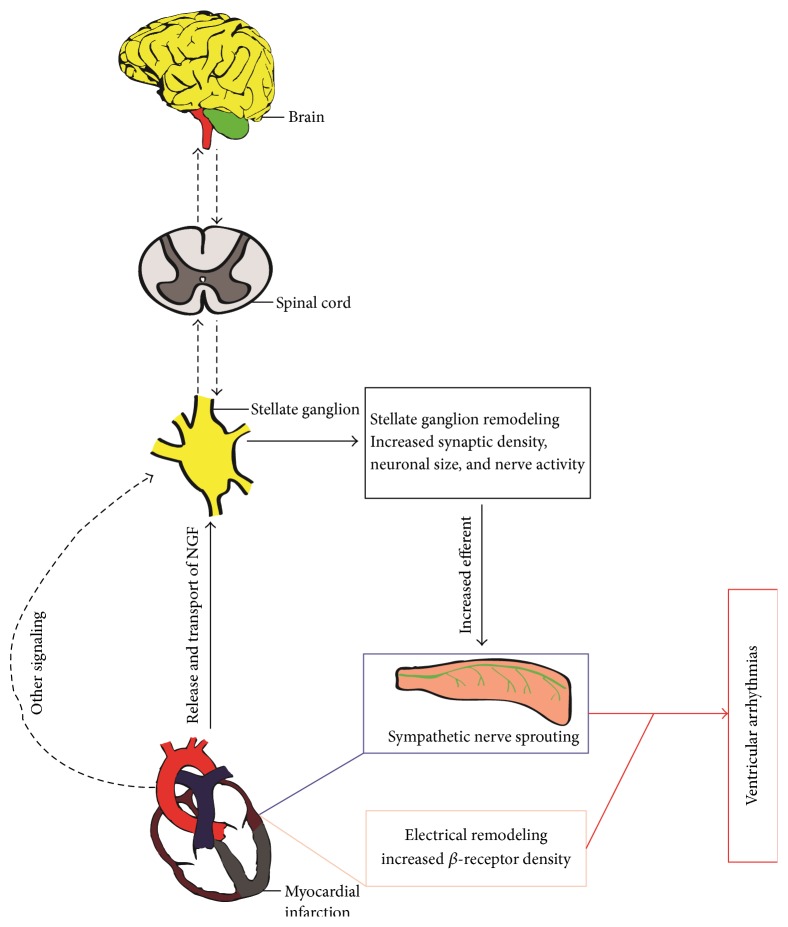
Sympathetic nerve remodeling increases the propensity for cardiac arrhythmias after myocardial infarction. Myocardial infarction induced NGF transported retrogradely to left (and right) stellate ganglion and other injury signals such as increased afferent nerve traffic lead to anatomic remodeling within the stellates. Increased efferent nerve signals back to the heart and promotes nerve sprout which together with electrical as well as *β*-adrenergic receptor remodeling increases the propensity for malignant ventricular arrhythmias. It is not clear whether higher nervous centers also regulate this process. Solid lines represent known pathways; dotted lines represent unknown.

It is widely accepted that sympathetic remodeling resulting from MI is strongly associated with the development of VT, VF, and SCD [1, 70, 71]. Indeed, evidence that the role of excessive cardiac sympathetic activity can directly precipitate VT has been provided by studies in patients and animal models with healed MI [72–74]. In contrast, interventions to decrease sympathetic nerve activity have been shown to provide a significant protection from arrhythmias in both patients and animals recovering from MI [75–77]. Importantly, the sympathetic nerve sprouting and compensatory reinnervation following MI have added an interesting dimension to arrhythmogenesis (Figure 2). Specifically, Cao et al. showed that native hearts of transplant recipients exhibit enhanced nerve fibers density around the diseased myocardium [24]. Significantly, an increased density of sympathetic nerves is higher in patients with a history of tachyarrhythmias than in those without tachyarrhythmias. Subsequently, to prove a causal relationship between sympathetic nerve sprouting and arrhythmogenesis, their group augmented myocardial nerve sprouting through chronic infusion of NGF [40] or continuous subthreshold electrical stimulation to the LSG [78]. In the experimental group, there was 2-fold increase of sympathetic nerve density and 10-fold increased incidence of VT compared with controls [40]. Moreover, four dogs that underwent NGF infusion died suddenly of spontaneous VF but none in the control group. Similarly, electrical stimulation to the LSG resulted in a dramatic increase of sympathetic nerve density and a much higher incidence of VT in dogs with MI and complete atrioventricular block (AVB) [78]. Cha et al. found that dogs with heart failure induced by rapid pacing exhibited increase of sympathetic nerve density, and dogs that died suddenly had greater nerve density [79], whereas our group found that attenuation of sympathetic nerve regeneration in the MI border zone by sema3A overexpression reduced the susceptibility to post-MI malignant arrhythmias and SCD [58]. All these data indicate a causal correlation between sympathetic nerve sprouting and arrhythmogenesis after MI.

The mechanisms, however, underlying this relationship remain incompletely understood. The excessive sympathetic nerves sprouting was closely correlated to increases of local ventricular transmural dispersion of repolarization [80] and prolongation of the QT interval [43], which may be a potential mechanism for fatal arrhythmia in chronic MI. In nerve-muscle coculture studies, investigators have reported sympathetic innervation may upregulate expression of functional L-type calcium channels [1]. Increased *I*_Ca_ may lengthen APD in myocardium. When *I*_Ks_ was downregulated and induced by MI, sympathetic stimulation tended to accentuate dispersion of repolarization [65]. Furthermore, several studies have shown that after MI, cardiac sympathetic hyperinnervation could modulate the expressions and functions of ion channels including *I*_K1_, *I*_to_ and ionotropic glutamate receptors (iGluRs) [68, 81, 82], leading to lengthening of the QT interval and increased dispersion of refractoriness, thereby resulting in the occurrence of VF and SCD. Thus deleterious sympathetic nerves sprouting might amplify the spatial heterogeneity of myocardial electrophysiological properties and underlie the occurrence of VT, VF, and SCD.

In addition, in ambulatory dogs with NGF to the LSG, AVB, and MI, most of the malignant ventricular arrhythmias were preceded by increased stellate ganglion nerve activity including low-amplitude burst discharge activity (LABDA) and high-amplitude spike discharge activity (HASDA), along with increased nerve sprouting [26]. Han et al. provided a direct physical evidence that structural neural remodeling was associated with increased sympathetic nerve activity after MI [83]. They described a persistent increase in the synaptic density of stellate ganglia accompanied by increased stellate ganglion nerve activity. It is possible that MI induced neurotrophic agents may be transported to the stellate ganglia via nerve tracts and then lead to remodeling of stellate ganglia as well as increase in stellate ganglion nerve activity followed by intramyocardial nerve sprouts [78, 84]. As increased sympathetic nerve discharge facilitates ventricular arrhythmias, hence, increased sympathetic ganglion nerve activity and nerves sprouting jointly contribute significantly to susceptibility to VF and SCD after MI.

It is well established that the sympathetic innervation of the heart functions mainly through the activation of *β*-adrenergic receptor (*β*-AR) by the release of norepinephrine (NE). In fact, Zhou and coworkers found that both deleterious nerve sprouting and significant increase in density of *β*3-AR occur in dogs with MI, AVB, and NGF infusion to the LSG [85]. *β*3-AR, presents in canine and human cardiac myocytes and exerts profound functions to mediate the membrane ion currents [86–88]. For example, activation of *β*3-AR could decrease slow delayed rectifier K^+^ current and *I*_Ks_, contributing to slight prolongation of the APD [87]. Additionally, Billman and coworkers have demonstrated dogs with healing MI susceptible to VF revealed a dramatically enhanced *β*2-AR response. Interestingly, similar responses did not occur before MI [89]. Presumably, *β*2-AR activation can provoke increase in intracellular Ca^2+^ transients and after contractions that ultimately trigger VF in the post-MI animal model [89, 90]. Although the nerve densities were not determined in those reports, there is overwhelming evidence MI can provoke nerve sprouting. Considering together these data, it is likely that *β*-AR remodeling in postinfarcted heart is important for formation of a substrate that triggers malignant ventricular arrhythmias and leads to SCD in chronic MI.

## 6. Conclusions

Collectively, excessive sympathetic nerve sprouting to reinnervation myocardium in response to MI may be an important element in the arrhythmogenicity of sympathetic nerves remodeling. On the other hand, modest nerve sprouting may contribute to improved hemodynamic performance of the surviving myocardium [91] and potent inhibition of nerve sprouting may also result in abnormal patterns of myocardial innervation and may facilitate propensity for the formation of malignant arrhythmias [52, 92]. In particular, significant advances have been made to understand the causal link between sympathetic nerve sprouting and VT, VF, and SCD after MI. The underlying mechanisms by which sympathetic nerve sprouting together with other changes such as electrical remodeling as a result of MI alter susceptibility to malignant arrhythmias are of considerable interest. Significantly, molecular mechanisms that regulate sympathetic nerves regeneration after MI remain largely to be determined. Better understanding of these complex problems may provide new tools for the prediction, prevention, and therapy of lethal arrhythmias after MI.
